# Real-World Characterization of Toxicities and Medication Management in Recipients of CAR T-Cell Therapy for Relapsed or Refractory Large B-Cell Lymphoma in Nova Scotia, Canada

**DOI:** 10.3390/curroncol32010002

**Published:** 2024-12-24

**Authors:** Jenna Shaw, Mahmoud Elsawy, Rachel Nielsen, Amye Michelle Harrigan, Tara T. DiCostanzo, Laura V. Minard

**Affiliations:** 1 Department of Pharmacy, Nova Scotia Health, QEII Health Sciences Centre, Halifax, NS B3H 2Y9, Canada; tarat.dicostanzo@nshealth.ca (T.T.D.); laurav.minard@nshealth.ca (L.V.M.); 2 QEII Health Sciences Centre, Division of Hematology and Hematologic Oncology, Dalhousie University, Halifax, NS B3H 4R2, Canada; mahmoud.elsawy@nshealth.ca (M.E.); amye.harrigan@nshealth.ca (A.M.H.); 3 Cell Therapy and Transplant Program, Nova Scotia Health, QEII Health Sciences Centre, Halifax, NS B3H 2Y9, Canada; rachel.nielsen@nshealth.ca

**Keywords:** CAR T-cell therapy, axicabtagene ciloleucel, large B-cell lymphoma, guideline adherence, guideline utilization, toxicity management

## Abstract

Nova Scotia (NS) began offering CAR T-cell therapy as a third-line standard of care for eligible patients with relapsed or refractory large B-cell lymphoma (r/r LBCL) in 2022. Recipients of CAR T-cell therapy often experience acute toxicities, including cytokine release syndrome (CRS) and immune effector cell-associated neurotoxicity syndrome (ICANS), which require close monitoring and prompt management. This retrospective review aimed to describe the characteristics of adult patients with r/r LBCL deemed eligible to receive CAR T-cell therapy with axicabtagene ciloleucel in NS between January 2022 and June 2024, the toxicities experienced and toxicity management, hospital visits and intensive care unit (ICU) admissions, the utilization of toxicity management guidelines, and general efficacy outcomes. Twenty-seven patients received axicabtagene ciloleucel. All patients experienced CRS (7.4% grade ≥ 3), and 55.6% developed ICANS (25.9% grade ≥ 3). The median hospital stay was 18 days, with 40.7% requiring ICU admission. There was one treatment-related mortality. Most CRS (85.2%) and ICANS (80.0%) cases were managed according to the guidelines. By day +100, the best objective response rate was 81.5% (44.4% complete responses). Patients who received CAR T-cell therapy in NS, Canada, experienced comparable toxicities and efficacy to those reported in pivotal clinical trials and other real-world experiences.

## 1. Introduction

Chimeric antigen receptor (CAR) T-cell therapy is a novel cancer immunotherapy that has revolutionized outcomes for patients with many hematological malignancies, including relapsed/refractory (r/r) large B-cell lymphomas (LBCL) [1,2,3]. Several clinical trials have demonstrated the curative potential of CAR T-cell therapy in patients with r/r LBCL [1,2,3,4,5]. The pivotal ZUMA-1 trial (n = 101) found that patients with r/r LBCL who received CAR T-cell therapy with axicel had an objective response rate (ORR) of 83% (58% complete responses), a median overall survival of 25.8 months, and a 5-year overall survival of 42.6% [1,5]. The ZUMA-1 trial demonstrates a significant improvement in outcomes compared to the historical benchmark results reported in the SCHOLAR-1 study, which was a large, international, multi-cohort pooled retrospective analysis that evaluated outcomes in patients with refractory diffuse large B-cell lymphoma (DLBCL) [6]. SCHOLAR-1 found consistently poor outcomes in this population, including an ORR of 26% (complete responses, 7%), median 6.3-month overall survival, and 20% survival after 2 years [6].

Despite the impressive and durable response rates seen with CAR T-cell therapy, potentially life-threatening toxicities can occur quickly, such as cytokine release syndrome (CRS) and immune effector cell-associated neurotoxicity syndrome (ICANS), which require close monitoring, appropriate grading, and prompt management [7,8,9,10]. Patients may require intensive care unit (ICU) admission for additional monitoring and interventions for high-grade CRS or ICANS, such as vasopressor administration, mechanical ventilation, or seizure management [7,8,9,10]. Hematotoxicity can also occur, with several studies reporting cytopenias after CAR T-cell infusion, including B-cell aplasia [1,11]. Cytopenias can be prolonged in some patients, posing a risk for infectious complications, which is the most frequent cause of nonrelapse mortality [11,12]. Additional toxicities include hypogammaglobulinemia, a consequence of B-cell aplasia, and nausea/vomiting [7].

**Figure A1:**
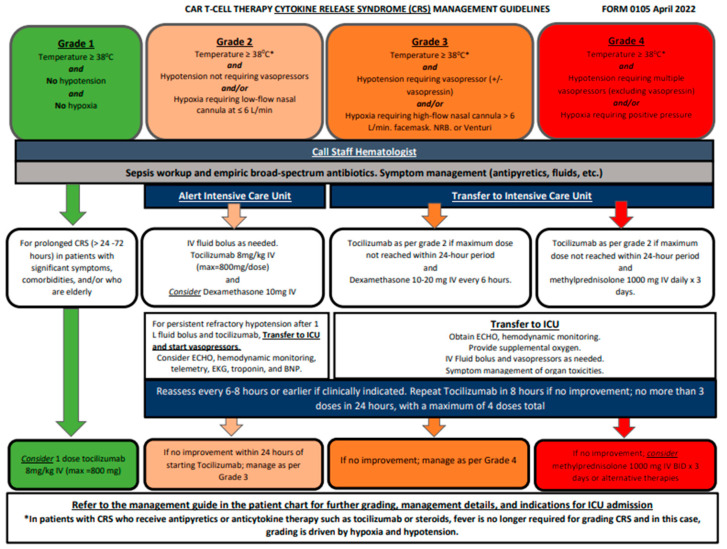
Nova Scotia Cell Therapy and Transplant Program (NSCTTP) CAR T-cell therapy cytokine release syndrome (CRS) management guidance document [25].

**Figure A2:**
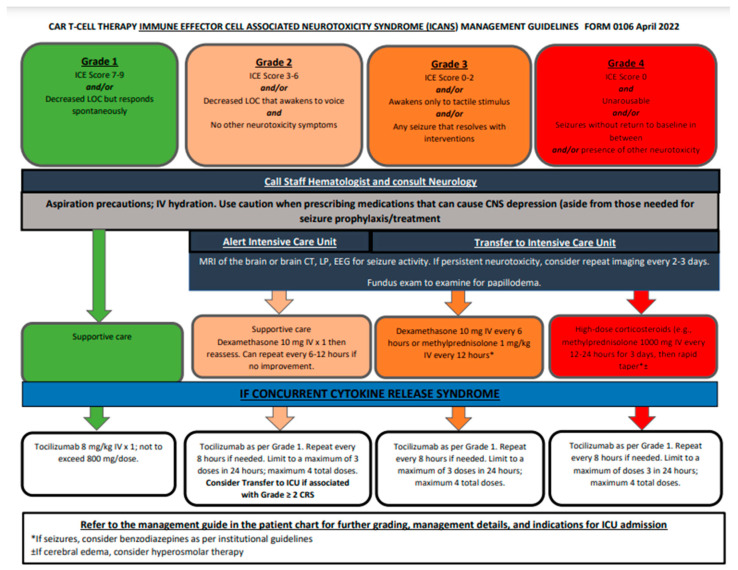
Nova Scotia Cell Therapy and Transplant Program (NSCTTP) CAR T-cell therapy immune effector cell-associated neurotoxicity syndrome (ICANS) management guidance document [25].

Several guidelines have been developed to assist clinicians with the recognition, grading, and management of CRS and ICANS. In general, CRS is managed with tocilizumab (an IL-6 receptor antagonist) alone or in combination with corticosteroids, whereas ICANS is typically managed with corticosteroids [8,10,13,14,15]. In NS, local guidance documents were developed as a collaborative effort between hematology and ICU teams, and the most recently published guidelines were adapted at that time (see Appendix A, Figure A1 and Figure A2).

Nova Scotia was the fourth Canadian province to offer CAR T-cell therapy with axicabtagene ciloleucel (axicel) as a third-line standard of care for patients with r/r LBCL. The first patient was treated in April 2022.

Given the common and serious toxicities associated with CAR T-cell therapy, it is important to characterize these toxicities and their management in real-world settings and to describe the utilization of toxicity management guidelines to facilitate the implementation of potential mitigation strategies to improve safety, as needed. It is also important to characterize the patients who have received CAR T-cell therapy, their general efficacy outcomes, and the associated utilization of healthcare system resources during the early phases of use in NS.

## 2. Materials and Methods

### 2.1. Study Design and Population

This was a retrospective chart review of adult (age ≥ 18 years) patients with r/r LBCL who were deemed eligible to receive CAR T-cell therapy with axicel in NS between January 2022 and June 2024. Patients were identified from NS Cell Therapy and Transplant Program (NSCTTP) records. Patients who received CAR T-cell therapy as part of a clinical trial were excluded due to differences in the treatment protocols and CAR T-cell products used; there were no patients who received axicel as part of a clinical trial during the study period. Patients were followed from the date they were deemed eligible for CAR T-cell therapy until the date of their last outpatient follow-up or the date of documented disease progression, whichever came first. Ethics approval was obtained from the Nova Scotia Health Research Ethics Board (REB# 1030118). The requirement for informed consent was waived by the board.

### 2.2. Objectives

The primary objectives of this study were to (a) describe the characteristics of patients approved to receive CAR T-cell therapy in NS, (b) characterize the toxicities associated with CAR T-cell therapy in our cohort and describe the management of these toxicities, (c) determine the number of hospital visits and ICU admissions by patients deemed eligible to receive CAR T-cell therapy in NS, (d) describe the utilization of toxicity management guidelines, and (e) describe general efficacy outcomes in patients who received CAR T-cell therapy in NS.

### 2.3. Data Collection

Data were collected using pharmacy and electronic health records. Patient demographics and characteristics were assessed prior to lymphodepleting chemotherapy. Details of the CAR T-cell therapy treatment were also collected. Efficacy outcomes were assessed using positron emission tomography (PET) scans and limited to results at day +30 and day +100 after CAR T-cell infusion. The results of the PET scans were categorized as either complete response (CR), partial response (PR), stable disease (SD), or disease progression (DP). The patients who had PR at day +30 had a repeat PET scan at day +60. To characterize the toxicities associated with CAR T-cell therapy, data on the occurrence of CRS, ICANS, cytopenias, hypogammaglobulinemia (defined as IgG < 4 g/L), infections, and nausea/vomiting were collected. For CRS and ICANS, severity, time to onset, duration, and management data were collected. The severity of CRS and ICANS was described using the American Society of Transplantation and Cellular Therapy (ASTCT) Consensus grading [16], and the severity of cytopenias and nausea/vomiting was graded using the Common Terminology Criteria for Adverse Events (CTCAE) version 5.0 [17]. Using CTCAE grading, severe cytopenias were that of grade three or higher [17]. Cytopenias were assessed daily from day +1 to day +30, and on day +100 for patients whose follow-up period was 100 days or more. The management of CRS and ICANS was categorized as being “managed according to guidelines” if the management aligned with at least one of five published guidelines [8,10,13,14,15] or the NS local guidance document (see Appendix A, Figure A1 and Figure A2). To describe hospital visits and ICU admissions, data were collected regarding the length of hospital stay, the length of ICU stay, the number of hospital readmissions after CAR T-cell therapy, and the number of outpatient visits with a hematologist or nurse practitioner after CAR T-cell therapy. The number of outpatient visits with a clinical pharmacist before and after CAR T-cell therapy was also determined, beginning in April 2023 when a new CAR T Pharmacy Clinic was implemented.

### 2.4. Statistical Analysis

Descriptive statistics were used for each outcome. For continuous variables, the results are reported as medians and ranges (e.g., time to onset of CRS). Frequencies and percentages are used to report the results for categorial variables (e.g., severity of CRS by grade).

## 3. Results

### 3.1. Patient Characteristics

A total of 32 patients were deemed eligible to receive CAR T-cell therapy with axicel in NS between January 2022 and June 2024. Of these patients, 27 were able to receive treatment and were treated with axicel. Of the five patients that did not receive axicel, all five had undergone apheresis. Three died prior to receiving axicel (two died due to infectious complications, and one died following lymphodepletion), and two opted not to receive further treatment. Patient baseline demographic and disease characteristics, as assessed prior to lymphodepleting chemotherapy, are shown in Table 1. The median age was 60.5 years (range 23–75), and 59.4% were male. Most patients were diagnosed with DLBCL (46.9%), and 71.9% of patients had refractory disease. Patients had received a median of two prior lines of therapy; 15.6% had received a previous autologous stem cell transplant. Almost half of the patients required bridging therapy (46.9%). Most patients had a baseline ECOG score of 0 (56.2%). All 27 patients treated with axicel received lymphodepleting chemotherapy with fludarabine and cyclophosphamide. All the patients received prophylactic therapies for tumor lysis syndrome (allopurinol) and seizures (levetiracetam) as well as antimicrobial prophylaxis (valacyclovir, fluconazole, sulfamethoxazole/trimethoprim, or dapsone, or pentamidine in cases of sulfa allergy). The median duration of follow-up was 230.5 days (range 28.5–709.5).

### 3.2. Toxicities

As shown in Table 2, all patients experienced CRS and 7.4% of cases were severe (i.e., grade ≥ 3). The median time to onset was 3 days (range 0 to 6) post-infusion, and the median duration was 4 days (range 1 to 8). Fifteen (55.6%) patients developed ICANS (25.9% grade ≥ 3). The median time to onset was 5 days post-infusion (range 1 to 10) and the median duration was 5 days (range 1 to 21). In total, 11 patients (40.7%) were admitted to the ICU. Six patients required ICU admission for CRS management and monitoring, two of whom required vasopressors, and five were admitted to the ICU for ICANS management and monitoring, two of whom experienced seizures. There was one treatment-related mortality: one patient died from bilateral pulmonary hemorrhage in the context of thrombocytopenia.

The medication management of toxicities is shown in Table 3. Tocilizumab was used to manage 25 of the 27 CRS cases (92.6%), with most patients receiving two doses (range 1 to 3). Corticosteroids were used in 17 patients (63.0%), three (17.6%) of whom were being treated for CRS alone, seven (41.2%) for ICANS alone, and the remaining seven for concurrent CRS and ICANS. Of the 17 patients who received corticosteroids for either CRS, ICANS, or both, 41.2% had a cumulative dose of less than 500 mg of prednisone equivalents, while three patients (17.6%) received a cumulative dose of greater than 3000 mg of prednisone equivalents. One patient received anakinra for steroid-refractory ICANS.

Severe neutropenia occurred in all patients (100%) prior to day +30, with 26 (96.3%) experiencing severe leukopenia prior to day +30. Data for cytopenias at day +100 were available for 16 patients: three remained severely neutropenic at day +100, and four remained severely leukopenic at day +100. Severe thrombocytopenia and anemia were less common before day +30. Two patients had severe thrombocytopenia at day +100, and one patient had severe anemia at day +100. The median time to the lowest ANC was 7 days after axicel (range 1 to 15), the median time to the lowest WBC was 5 days (range 2 to 7), and the median time to the lowest platelet count was 7 days (range 1 to 304).

Fourteen (51.9%) patients experienced hypogammaglobulinemia, and nine (33.3%) received intravenous immunoglobulin (IVIG). Infection occurred in 14 patients (51.9%), with bacterial infections being the most common—ten patients (37.0%) experienced at least one bacterial infection. Viral infections were diagnosed in five patients (18.5%) and fungal infection in one patient (3.7%).

Nausea and/or vomiting occurred in nearly half of all patients (48.1%), with one case graded as severe (3.7%). Most cases of nausea and/or vomiting presented early after CAR T-cell infusion, with a median time to onset of 3.5 days (range 1 to 15).

### 3.3. Hospital Visits and ICU Admissions

The median length of stay in hospital was 18 days (range 10 to 47). Among the 11 patients (40.7%) that were admitted to the ICU, the median length of stay was 31.4 h (range 10.7 to 96.0). Once discharged from hospital and, subsequently, from the outpatient day unit, the patients had a median of five outpatient visits (range 0 to 18) with a physician or nurse practitioner. Six patients (22.2%) were readmitted to hospital during the follow-up period: three due to COVID-19 infection, two for organizing pneumonia, and one for ongoing ICANS. From the time of approval for CAR T-cell therapy to the end of the follow-up period, the patients had a median of three (range one to four) outpatient visits with a clinical pharmacist.

### 3.4. Utilization of Toxicity Management Guidelines

When looking strictly at the wording in the CRS and ICANS management guidelines and without considering the prescriber’s clinical judgment of patient factors that warrant management that differs from guideline recommendations, 23 (85.2%) CRS cases were managed according to at least one management guideline [8,10,13,14,15]. We identified four cases (14.8%) that were managed differently from the guidelines. In two cases, tocilizumab was administered earlier than the recommended 24 h after the onset of grade 1 CRS. Specifically, in two cases, tocilizumab was initiated less than 10 h after the onset of grade 1 CRS in the absence of progression to grade 2. In one of these cases, CRS progressed to grade 3 within 24 h of tocilizumab administration, and in the other case, CRS did not progress beyond grade 1 and was resolved within 3 days. In another case, there were 6 h rather than the recommended 8 h between tocilizumab doses; in this case, CRS did not progress further and was resolved after 3 days. In a fourth case, corticosteroids were used to manage grade 1 CRS, and at the time of data collection, the guidelines recommended corticosteroids to manage grade ≥ 2 CRS. In this case, CRS did not progress beyond grade 1 and was resolved after 5 days. In the 15 patients who experienced ICANS, 12 (80.0%) were managed according to at least one guideline. Three cases were managed differently from the guidelines: the corticosteroid dose was lower than that recommended in two cases (8 mg of dexamethasone was prescribed rather than 10 mg), and corticosteroid was started at the onset of grade 1 ICANS in one case. In the cases where the corticosteroid dose was lower than that recommended, they did not progress beyond grade 2 ICANS, and both cases resolved within 24 h. In the case where corticosteroid was started at the onset of grade 1 ICANS, severity had progressed to grade 2 within 2 h of receiving corticosteroid. All patients fully recovered from CRS and/or ICANS, including those cases where the management was different from the guidelines.

### 3.5. Efficacy Outcomes

**Figure 1:**
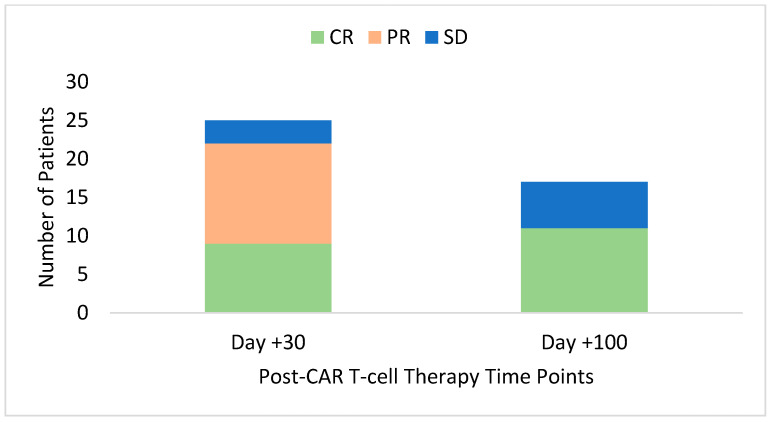
Stacked bar graphs showing the rates of complete response (CR), partial response (PR), and stable disease (SD) based on positron emission tomography (PET) scans at day +30 and day +100 in patients who received CAR T-cell therapy with axicabtagene ciloleucel in Nova Scotia.

The efficacy outcomes, shown in Table 4 and Figure 1, are limited to day +100 post-CAR T-cell infusion. By day +30, nine patients (33.3%) had achieved CR: eight maintained their response at day +100, and one died prior to day +100. Thirteen (48.1%) patients achieved a PR by day +30, three of which had changed to CR by day +100. Other patients who achieved PR at day +30 remained stable or developed DP. For patients with SD at day +60, this was maintained at day +100. Two patients had DP by day +30, and five had DP by day +60. By day +100, the best objective response rate (ORR) was 81.5% (44.4% complete responses), with 40.7% remaining in CR at day +100.

## 4. Discussion

This is the first real-world characterization of CAR T-cell therapy recipients in NS, and to our knowledge, the third real-world study of patients receiving axicel in Canada [18,19]. One published study included 15 patients who received axicel for r/r LBCL in the province of Quebec [18], while the other is a conference abstract that reported on 27 patients who received axicel for r/r LBCL in Ontario, another Canadian province [19]. Our study included 27 patients with r/r LBCL who were treated with axicel between January 2022 and July 2024 in NS, Canada. We also characterize the toxicities experienced by patients receiving CAR T-cell therapy, including how these toxicities were managed, and our assessments of (1) hospital visits and ICU admissions and (2) the utilization of CRS and ICANS toxicity management guidelines add novel findings to the literature. We report a CR rate of 40.7% at day +100, which is comparable to the results from clinical trials and another real-world Canadian study that found a CR rate of 33% at day +90 [1,18].

Consistent with findings from early clinical trial data [1,4,20] and another real-world Canadian study by Benoit et al. [18], all patients in our study developed CRS. Our rate of severe CRS (7.4%) is similar to the rate of severe CRS (6%) reported in ZUMA-7 (axicel in a second-line setting) and lower than that reported in ZUMA-1 (13%) [1,4]. Of note, the rate of severe CRS in ZUMA-1 cohort 4, which included the earlier pharmacological management of CRS, was only 2% [20]. A study by Jacobson et al. assessing the real-world efficacy and safety of axicel for r/r LBCL in 122 patients across the United States found that 16% developed CRS of grade 3 or higher [21]. A later study by Jacobson et al. that assessed the efficacy and safety of axicel in 1297 patients with r/r LBCL found that 83% developed CRS, with 8% experiencing CRS of grade 3 or higher [22]. It is important to note that comparisons of rates of toxicities between studies are limited by the use of different grading systems. Although the proportion of patients who received tocilizumab in our study (92.6%) is comparable to the findings from Benoit et al. (87%), it is higher than the 66% and 58% reported in the 2020 and 2022 studies by Jacobson et al. [18,21,22]. Our proportion of patients treated with corticosteroids for CRS alone and for concurrent CRS and ICANS is similar to the 12.3% and 30.8% reported by Jacobson et al., respectively [21]. Of note, recent guidelines have trended toward the earlier medication management of CRS and ICANS, and strategies to mitigate these toxicities will continue to evolve over time [20].

Our rates of ICANS are similar to those seen in ZUMA-1 (64% any grade, 28% severe) [1] and those reported by Jacobson et al. (55% any grade, 24% severe) [22]; however, we observed higher rates compared to Benoit et al. (40% any grade, 13% severe) [18].

Cytopenias, including anemia, thrombocytopenia, and neutropenia, are reported in approximately 30–80% of patients who receive CAR T-cell infusion [1,4]. Real-world data reports cytopenias lasting longer than 28 days in 47.6% of patients [23], which is higher than what is reported in this study; however, interpretation is limited, as day +100 data were only available for 16 patients.

Hypogammaglobulinemia occurred in 14 patients (51.9%) post-infusion, and 9 patients (33.3%) received prophylactic IVIG. Given the slower onset of hypogammaglobulinemia, data were not yet available for all patients at the time of this report.

Toxicities associated with CAR T-cell therapy, namely cytopenias and hypogammaglobulinemia, can significantly increase the risk for infectious complications [8,10]. In our cohort, most infections that occurred were bacterial, while viral and fungal infections were less common. This reflects the pattern of early infections observed post-CAR T-cell therapy [12].

The rates of nausea/vomiting observed in our study population are lower (48.1%) than those reported in ZUMA-1 (58%) [1]. Although our sample size was much smaller, it is possible that we observed less nausea/vomiting due to differences in antiemetic prophylaxis. The need to avoid corticosteroids in the days leading up to CAR T-cell infusion [24] creates a challenge to the prevention of chemotherapy-induced nausea and vomiting in this patient population. Antiemetic prophylaxis for recipients of CAR T-cell therapy in NS originally included ondansetron and olanzapine; however, this has since been optimized to an NK1-receptor antagonist and 5HT_3_ receptor antagonist (netupitant and palonosetron) in recognition of the emetogenic potential of cyclophosphamide and fludarabine in the absence of dexamethasone utilization.

After hospital discharge, patients were followed closely with outpatient visits with physicians, nurse practitioners, and pharmacists. Readmission to hospital due to infectious complications occurred in approximately one-fifth of patients during the follow-up period, including one patient who was readmitted for toxicity management. Jacobson et al. report that ICU transfer occurred in 28% of their cohort for toxicity management, and 18% required readmission to hospital for toxicity management [22]. There is limited data available to compare hospital visits and ICU admissions among CAR T-cell therapy recipients at different centers.

We are not aware of any reports describing the utilization of CAR T-cell therapy toxicity management guidelines for CRS and ICANS. When compared strictly to the wording in CRS and ICANS management guidelines, most cases of CRS and ICANS were managed according to at least one guideline [8,10,13,14,15]. The most common reason for deviation from the guidelines was starting tocilizumab earlier than recommended for CRS or slightly different corticosteroid dosing than that recommended in the guidelines. Given these subtle differences in corticosteroid dosing, the creation of an order set to guide corticosteroid dosing by grade for CRS and ICANS may facilitate the ability of the prescriber to select a standardized dose where appropriate. Since toxicity management recommendations are rapidly evolving, order sets would need to be updated as new evidence comes to light.

Importantly, some cases where management was different from guideline recommendations may not be clinically significant (e.g., use of 8 mg of dexamethasone instead of 10 mg). When looking at CRS and ICANS cases where management differed from guideline recommendations, we found that some cases progressed in severity despite earlier management. It is important to consider that, in certain cases, differences in management from the guidelines are warranted, depending on the patient’s condition and the prescriber’s clinical judgment. Clinical decisions are dynamic and not always documented in the patient chart, making it difficult to assess the reasoning behind specific CRS and ICANS management strategies in cases where this varied from guideline recommendations. Our assessment of guideline utilization highlights the nature of these dynamic clinical situations where individualized decisions are made integrating baseline risk factors and clinical judgment.

This study is limited by its retrospective design and small sample size. Since this was a chart review, missing and/or inaccessible documentation may have resulted in an underestimation of toxicities, especially those occurring in the outpatient setting (e.g., infection). Of note, the symptoms of CRS and ICANS were only assessed at the time of onset and at the highest grade; we did not determine whether management was in accordance with the guidelines at other time points. The reasons for managing toxicities outside guideline recommendations were not evaluated and may have been due to intentional clinical decision-making by the physician or other members of the healthcare team. Additionally, this study did not account for changing guidelines over time. As the CAR T-cell therapy program in NS expands, future studies with a longer follow-up period, larger sample size, or a multicenter design could help to overcome some of these limitations and give insight to longer-term efficacy and safety outcomes. It would also be useful to evaluate reasons for managing toxicities differently from guideline recommendations. Additional research could focus on patients who receive other CAR T products and/or patients with other indications for CAR T-cell therapy.

## 5. Conclusions

This study provides novel real-world insights into the early experience of CAR T-cell therapy in NS, Canada. Overall, the patient outcomes and rates of toxicities were comparable to those reported elsewhere, and the majority of CRS and ICANS cases were managed according to clinical practice guidelines. With the increasing availability of CAR T-cell therapy for patients across Canada and internationally, we expect CRS and ICANS management guidelines will continue to evolve as new evidence comes to light. Findings from this study can be used when planning for the expansion of the CAR T-cell therapy program in Nova Scotia. The high incidence of toxicities, including CRS and ICANS, and the need for close monitoring highlight the need for adequate staffing, particularly in the inpatient setting. A low nurse-to-patient ratio in the initial post-infusion period when patients are at the highest risk of severe complications will facilitate patient safety. Similarly, bed allocation planning must account for the prolonged hospital stays observed in many patients as well as ICU resource availability for those with severe toxicities. Developing dedicated CAR T-cell therapy units with appropriately trained staff could ensure timely intervention and streamline care delivery. Additionally, insights from this study highlight the importance of outpatient resources to monitor and manage delayed complications, which could reduce readmissions and improve overall healthcare efficiency.

## Figures and Tables

**Table 1:** Baseline characteristics of patients deemed eligible to receive CAR T-cell therapy (n = 32) and patients who received CAR T-cell therapy with axicabtagene ciloleucel (n = 27) in Nova Scotia between January 2022 and June 2024.

Characteristic	Patients Deemed Eligible for CAR T-Cell Therapy, n (%) n = 32	Patients Who Received CAR T-Cell Therapy, n (%) n = 27
**Age ^a^ (median, range)**	60.5 (23–75)	60.0 (23–75)
**Male sex**	19 (59.4)	13 (48.1)
**Diagnosis**		
DLBCL	15 (46.9)	12 (44.4)
tFL	10 (31.2)	9 (33.3)
High-grade LBCL	7 (21.9)	6 (22.2)
**Relapsed vs. refractory disease**		
Refractory disease	23 (71.9)	19 (70.4)
Relapsed disease	9 (28.1)	8 (29.6)
**Previous therapies**		
Number of prior lines of therapy (median, range)	2 (2–6)	2 (2–6)
ASCT	5 (15.6)	3 (11.1)
**ECOG performance status** ^b^		
0	18 (56.2)	17 (63.0)
1	11 (34.4)	8 (29.6)
2	3 (9.4)	2 (7.4)
**Bridging therapy**		
No bridging therapy	17 (53.1)	14 (51.9)
Pola-BR	9 (28.1)	7 (25.9)
Other ^c^	6 (18.8)	6 (22.2)
**Lymphodepleting chemotherapy**		
Fludarabine/Cyclophosphamide	27 (84.4)	27 (100.0)

^a^ Age at the time of leukapheresis, ^b^ the last documented ECOG Performance Status between the time of eligibility for CAR T-cell therapy and the time of lymphodepleting chemotherapy, ^c^ corticosteroids and/or radiation therapy. Abbreviations: ASCT, autologous stem cell transplant; CAR, chimeric antigen receptor; DLBCL, diffuse large B-cell lymphoma; ECOG, Eastern Cooperative Oncology Group; LBCL, large B-cell lymphoma; Pola-BR, polatuzumab/bendamustine/rituximab; tFL, transformed follicular lymphoma.

**Table 2:** Toxicities associated with CAR T-cell therapy among patients in Nova Scotia who received axicabtagene ciloleucel between January 2022 and June 2024 (n = 27).

Toxicity	
**CRS—** **n (%)**	27 (100)
Grade ≥ 3—n (%)	2 (7.4)
Median time to onset—days (range)	3 (0–6)
Median duration—days (range)	4 (1–8)
ICU admission—n (%)	6 (22.2)
ICU median duration—hours (range)	24.5 (10.7–77.3)
**ICANS—** **n (%)**	15 (55.6)
Grade ≥ 3—n (%)	7 (25.9)
Median time to onset—days (range)	5 (1–10)
Median duration—days (range)	5 (1–21)
ICU admission—n (%)	5 (18.5)
ICU median duration—hours (range)	35.3 (19.1–22.4)
**Neutropenia ^a^—** **n (%)**	27 (100)
Prior to day +30—n (%)	27 (100)
At day +100—n (%)	3 (11.1)
**Leukopenia ^b^—** **n (%)**	26 (96.3)
Prior to day +30—n (%)	26 (96.3)
At day +100—n (%)	4 (14.8)
**Thrombocytopenia ^c^—** **n (%)**	11 (40.7)
Prior to day +30—n (%)	11 (40.7)
At day +100—n (%)	2 (7.4)
**Anemia ^d^—** **n (%)**	10 (37.0)
Prior to day +30—n (%)	10 (37.0)
At day +100—n (%)	1 (3.7)
**Hypogammaglobulinemia ^e^—** **n (%)**	14 (51.9)
Median time to onset—days (range)	38 (30–231)
IVIG prophylaxis—n (%)	9 (33.3)
**Infection ^f^—** **n (%)**	14 (51.9)
Bacterial—n (%)	10 (37.0)
Viral—n (%)	5 (18.5)
Fungal—n (%)	1 (3.7)
**Nausea/Vomiting—** **n (%)**	13 (48.1)
Grade ≥ 3—n (%)	1 (3.7)

^a^ Severe neutropenia (absolute neutrophil count < 0.50 × 10^9^ cells/L); ^b^ severe leukopenia (white blood cell count < 2.0 × 10^9^ cells/L); ^c^ severe thrombocytopenia (platelet count < 50 × 10^9^ cells/L); ^d^ severe anemia (hemoglobin < 80 g/L); ^e^ hypogammaglobulinemia (IgG < 4 g/L); ^f^ in total, 14 patients experienced 16 infections. Abbreviations: CRS, cytokine release syndrome; ICANS, immune effector cell-associated neurotoxicity syndrome; ICU, intensive care unit; IVIG, intravenous immunoglobulin.

**Table 3:** Drug therapies used as part of toxicity management in patients who received axicabtagene ciloleucel in Nova Scotia between January 2022 and June 2024 (n = 27).

Drug Therapy	
**Tocilizumab—n (%)** ^a^	25 (92.6)
Median number of doses per patient (range) ^b^	2 (1–3)
**Corticosteroids—n (%)** ^a^	17 (63.0)
CRS alone—n (%) ^c,d^	3 (17.6)
ICANS alone—n (%) ^c,d^	7 (41.2)
CRS and ICANS—n (%) ^c,d^	7 (41.2)
Mean cumulative dose—mg ^c,e^	800.4
Dose ≤ 500 mg—n (%) ^c,e^	7 (41.2)
Dose > 500 mg and ≤1000 mg—n (%) ^c,e^	3 (17.6)
Dose > 1000 mg and ≤3000 mg—n (%) ^c,e^	4 (23.5)
Dose > 3000 mg—n (%) ^c,e^	3 (17.6)
Median duration—days (range) ^c,f^	4 (1–20)
**Broad-spectrum antibiotics—n (%)** ^a,g^	27 (100.0)
Median duration—days (range) ^a,h^	12 (5–30)
Anakinra—n (%) ^a^	1 (3.7)
Vasopressors—n (%) ^a^	2 (7.4)

^a^ n = 27, ^b^ n = 25, ^c^ n = 17, ^d^ reason for prescribing, ^e^ prednisone equivalents, ^f^ total number of days on which corticosteroids were received, ^g^ piperacillin/tazobactam, meropenem, ^h^ total number of days on which antibiotics were received. Abbreviations: CRS, cytokine release syndrome; ICANS, immune effector cell-associated neurotoxicity syndrome.

**Table 4:** Efficacy outcomes at day +30, day +60, and day +100 post-CAR T-cell infusion, as identified by PET scans in patients who received axicabtagene ciloleucel in Nova Scotia between January 2022 and June 2024 (n = 27).

Response	Day +30	Day +60	Day +100
Complete Response—n (%)	9 (33.3)	0 (0.0)	11 (40.7)
Partial Response—n (%)	13 (48.1)	2 (7.4)	0 (0.0)
Stable Disease—n (%)	3 (11.1)	6 (22.2)	6 (22.2)
Disease Progression—n (%)	2 (7.4)	5 (18.5)	1 (3.7)

Abbreviations: CAR, chimeric antigen receptor; PET, positron emission tomography.

## Data Availability

The original contributions presented in this study are included in the article. Further inquiries can be directed to the corresponding author.
